# *Yarrowia lipolytica* and *Lactobacillus paracasei* Solid State Fermentation as a Valuable Biotechnological Tool for the Pork Lard and Okara’s Biotransformation

**DOI:** 10.3390/microorganisms8081098

**Published:** 2020-07-22

**Authors:** Mihaela Cotârleț, Nicoleta Stănciuc, Gabriela Elena Bahrim

**Affiliations:** Department of Food Science and Engineering, Biotechnology and Aquaculture, Faculty of Food Science and Engineering, Dunărea de Jos University, 800201 Galaţi, Romania; mihaela.cotarlet@ugal.ro (M.C.); nicoleta.stanciuc@ugal.ro (N.S.)

**Keywords:** okara, pork fat, *Yarrowia lipolytica*, *Lactobacillus paracasei*, solid state fermentation, antioxidant activity, antimicrobial activity

## Abstract

This study reports the biovalorization of the two agri-food by-products (pork lard and freeze-dried okara) through solid-state fermentation using a monoculture of *Yarrowia lipolytica* or a co-culture of *Y. lipolytica* and *Lactobacillus paracasei*, for developing a valuable fermented product with antioxidant and antimicrobial activity. First, some yeast strains were selected based on their properties to produce enzymes (protease and lipase) by cultivation on 5% (*w*/*v*) pork lard or 2% (*w*/*v*) freeze-dried okara. Two selected strains, *Y. lipolytica* MIUG D5 and *Y. lipolytica* ATCC 18942, were further used for the fermentation alone or in a co-culture with *L. paracasei* MIUG BL2. The Plackett–Burman experimental design was used to establish the effects of the fermentation parameters in order to obtain a fermented product with improved antioxidant and antimicrobial activities. As the Plackett–Burman experimental design are independent variables, the concentrations of the freeze-dried okara, pork lard, glycerol, inoculums type, inoculum concentration, and the fermentation time were analyzed. The 2,2-diphenyl-1-picrylhydrazyl (DPPH) radical scavenging potential and the antimicrobial activity against aerobic spore-forming microorganisms were assessed as responses. For the fermented products, an antioxidant potential between 6.77–17.78 mM TE/g was obtained while the antimicrobial activity against *Aspergillus niger* ranged from 24 to 64%. Based on the statistical analysis, the time of the yeast fermentation and the concentration of pork lard were selected as variables with the influence on the SSF fermentation process and the functional properties of the fermented product. In the general context of a circular economy, the results demonstrate the possibility of bio-transforming the freeze-dried okara and the pork lard using *Y. lipolytica* as a valuable workhorse for the lactic acid bacteria (LAB) metabolism and postbiotics production into a fermented product, which is recommended for use as a food and feed ingredient with biotic properties.

## 1. Introduction

Okara is a food processing by-product derived from soybeans (*Glycine max*), being the solid residue that remains after filtering the water-soluble fraction during soymilk or soybean curd (tofu) production [1]. About 1.2 kg of okara is obtained from every 1 kg of soybeans that are used in the tofu production [2]. Therefore, large amounts of okara are produced annually, especially in Asian countries with high soybean consumption. The amount of okara produced by the tofu manufacturing sector is about 800,000 t in Japan; 310,000 t in Korea; and 280,0000 t in China. Even with the large volumes of okara generated by the food industry, most of it is discarded due to the okara’s high moisture content, which makes it very perishable [3,4]. This by-product is a rich source of high-quality proteins, carbohydrates, unsaturated lipids, and dietary fiber, and also contains isoflavones, minerals, and oligosaccharides [4]. Therefore, okara is a potential inexpensive functional food ingredient [5]. However, its rate of reuse is still low and is intended almost exclusively for animal feed [2,6] or human food, with some limitations due to its oligosaccharide content, which produces flatulence and the disagreeable ‘fishy’ and ‘beany’ flavor, whereas the rest is discarded as industrial garbage [1].

Annually, in Europe, countries like Germany, France, the UK, and Spain are processing approximately 16 million tons of animal fat by-products [7]. The pork lard was associated for a long time as a potential cause of vascular and heart disease [8] and therefore not used as much in food preparation. The price of animal fat is relatively low, which makes it a cheap and promising substrate for microbial fermentation [9] in order to obtain valuable metabolites (i.e., short- and medium-chain fatty acids) [10].

Solid-state fermentation (SSF) is a conventional fermentation method that may be designed to improve the nutritional value and functional properties of food processing by-products [1]. The biological activities of okara can be improved by SSF due to the fact that the microbial proteases are able to metabolize the high molecular weight proteins to smaller, bioactive peptides or amino acids. Additionally, the microbial lipase can metabolize the pork fat in order to generate the valuable fatty acids. Okara was used as a fermentation substrate to produce a variety of products for human consumption including α-glucosidase inhibitors that are used in the diabetes mellitus treatment, β-fructofuranosidase, fibrinolytic enzymes, iturin A, edible fungi, chitosan, alcohol, and biosurfactants [4]. Additionally, okara can support probiotic growth and survival in different formulated media and under simulated gastrointestinal conditions [5]. Additionally, Vong and Liu (2016) [3] reported a study that regards the fermented okara with improved nutritional value, its aroma compounds, and its high antioxidant potential.

*Yarrowia lipolytica* is an excellent microorganism with multiple biotechnological applications [11]. On the other hand, lactic acid bacteria (LAB), belonging to the *Lactobacillus* spp. genus., are considered important in relation to the biodegradation of fatty materials, thus enhancing the digestibility of proteins and supporting human health by inhabiting the gastrointestinal tract. These properties of LAB are attributed to their metabolic processes including enzymes such as lipases, proteases, and antibacterial proteins [12]. Both *Y. lipolytica* and *Lactobacillus* spp. are used in the production of fermented products due to their generally recognized as safe (GRAS) status [6,13]. Fermentation using microorganisms in a co-culture such as LAB and yeast could provide promising results such as an increase in the microbial safety status of the products by reducing the potential spore forming microorganism [4] due to the production of organic acids and other inhibitory substances [3]. Thus, due to their strong metabolic activity and diversity, the co-fermentation of yeast and lactobacilli offers great potential for the biotransformation of okara and pork lard. However, to the best of our knowledge, the fermentation of both okara and pork lard in a yeast and lactobacilli co-culture has not been investigated previously. Instead, Tzirita et al. (2018) [10] reported on waste fat biodegradation by *Y. lipolytica*, *Bacillus* spp. and *Pseudomonas putida* consortium in order to produce lipids with significant industrial, ecological, and biotechnological interest.

The aim of this study was to investigate the microbial transformation of the freeze-dried okara and pork lard as a valuable biotechnological way for the economically attractive production of value-added, functional products for food and feed applications by using a co-culture of *Y. lipolytica* and *L. paracasei*. First, the selection of the yeast strains based on their capacity to biosynthesize lipases and proteases was performed. Second, different microbial consortia for solid state fermentation of the substrates were tested. Furthermore, the identification of the parameters that influence the SSF fermentation in order to obtain a fermented product with improved antioxidant and antimicrobial potential was achieved by applying the Plackett–Burman experimental design.

## 2. Materials and Methods

### 2.1. Materials and Microorganisms

Soybeans not genetically modified were purchased from Solaris Plant S.R.L., Bucharest, Romania. The okara was obtained through the method described by Vong and Liu (2016) [3] with some modifications. The grains were hydrated with tap water in a ratio of 1:6 (*w*/*v*), for 16 h at 4 °C, followed by grinding for 10 min and filtering. The solid by-product was lyophilized using the Alpha 1–4 equipment (Martin Christ, Osterode am Harz, Germany), followed by grinding. The resulting powder contained 27.3% carbohydrates, 26.4% proteins, 21.7% dietary fiber, 18.3% lipids, and 4.3% ash. The pork lard was procured from a local artisanal farm.

Nineteen yeast strains of *Y. lipolytica*, *Candida lipolytica*, *C. krusei*, and *C. colliculosa* and one strain of *L. paracasei* were used. These strains are part of the Collection of Microorganisms (acronym MIUG) of Bioaliment Research Platform from the Faculty of Food Science and Engineering, “Dunărea de Jos” University, Galați, Romania. One of the yeast strains, *Y. lipolytica* ATCC 18942, was purchased from the American Type Culture Collection (ATCC), Manassas, VA, USA.

To test the antimicrobial activity, *Aspergillus niger* MIUG M5 and *Bacillus subtilis* MIUG B1 strains were used as indicator microorganisms. All the chemicals, reagents, and commercial culture media were purchased from Sigma-Aldrich (Steinheim, Germany).

Bottom of Form.

### 2.2. Selection of the Yeast Strains as Lipase and Protease Producers

The *Y. lipolytica*, *C. lipolytica*, *C. krusei*, and *C. colliculosa* yeast strains were studied for their capacity to produce lipases and proteases. To underline the lipase activity, the following selective media was used (*w*/*v*%): pork lard 5.0, peptone 0.5, yeast extract 0.25, agar 2.0, and pH 6.5. Sequentially, to detect the proteolytic strains, the following medium was used (*w*/*v*%): freeze-dried okara 2.0 and agar 2.0, pH 6.5.

To assess the hydrolases (protease and lipase) activity assay, the radial diffusion method was used.

Therefore, for the lipase activity, the specific media was supplemented with 1% Rhodamine B (1 mg/mL) and inoculated with the yeast cells [14]. After incubation at 28 °C for five days, an orange fluorescent halo visible upon UV irradiation (Ĉ = 350 nm) around the active yeast colonies appeared [15]. The colony diameter was measured and expressed in mm.

Furthermore, the lipolytic activity of the most active strains of *Y. lipolytica* was assayed by the plate diffusion method, in the spirit blue agar medium (Difco), according to the method of Parfene et al. (2012) [16] with some modifications. The rehydrated commercial media in accordance with the manufacturer was supplemented with 2% pork lard. The pH of the media was adjusted to 5.0, sterilized at 121 °C for 15 min, and was then centrally inoculated with the yeast biomass. The growth was performed at 28 °C for 48 h. The lipolysis was assessed by observing the halos on the plate, which in turn indicated that the selected microorganisms metabolized the lipids [17]. The hydrolysis index (HI) was determined as the ratio between the diameter of the hydrolysis zone and the diameter of the colony.

Regarding the protease activity, after the incubation at 28 °C for five days, the most active strains were highlighted through the appearance of a clear zone around the colonies, compared to the rest of the medium that was opaque [18]. The HI was also taken into account.

### 2.3. Inoculum Preparation and Solid State Fermentation (SSF) Fermentation

All the stock cultures were preserved in 40% glycerol at −80 °C. The yeast strains were reactivated on a yeast extract peptone dextrose (YPD) agar medium for 3–5 days, at 28 °C. In order to obtain the inoculum, the yeast biomass was transferred into 50 mL sterile physiological solution (0.9% NaCl). Then, the yeast cells were counted with the Thoma cytometer and inoculated in the fermentation medium at a final concentration of 1.0 × 10^7^ CFU/g.

The LAB culture was reactivated on De Man, Rogosa and Sharpe broth (MRS) for 48 h at 37 °C. A 2% inoculum with an optical density (OD) of 2.601 measured at the wavelength of 600 nm with a UV/VIS V-530 spectrophotometer (Jasco, Tokyo, Japan).

#### 2.3.1. SSF Fermentation with the Monoculture of *Y. lipolytica*

The cultivation in the SSF system was prepared in sterile glass Petri dishes with a diameter of 120 mm. Thus, 10.0 g of freeze-dried okara, 2.0 g of pork lard, 1.0 g of glycerol, and 15 mL of basal medium consisting of 0.7 KH_2_PO_4_, 0.25 Na_2_HPO_4_, 0.15 MgSO4 × 7H_2_O (w/v%), and pH 7.0 were sterilized at 121 °C for 15 min. After cooling, the medium was inoculated with 1 mL of yeast inoculum to obtain a final concentration of 1.0 × 107 CFU/g. The fermentation took place in a stationary system at 30 °C for five days. Two fermented products were developed, one with *Y. lipolytica* MIUG D5 and one with *Y. lipolytica* ATCC 18942.

#### 2.3.2. SSF Fermentation with the Co-Culture of *Y. lipolytica* and *L. paracasei*

The yeast cultivation in a SSF system was prepared as described above with the mention that the yeast was cultivated for 72 h at 30 °C. Then, 2% of the *L. paracasei* MIUG BL20 inoculum was added and incubated further for another 72 h. Two fermented products were developed, one with the co-culture of *Y. lipolytica* MIUG D5 and *L. paracasei* MIUG BL20 and the other one with *Y. lipolytica* ATCC 18942 and *L. paracasei* MIUG BL20.

At the end of the fermentation, the fermented medium was subjected to freeze-drying and then tested to assess the antioxidant and antimicrobial activity.

### 2.4. Identifying the Significant Variables of the Biotechnological Process by Using the Plackett–Burman Design

The Plackett–Burman experimental design was used as an effective tool for the screening of the fermentation parameters with respect to their main effects on the process. The variables chosen for the present study were the freeze-dried okara, pork lard, glycerol concentration, yeast, and LAB inoculum concentration, and the fermentation time. As responses, the antioxidant and antimicrobial activities of the fermented products were considered. The experimental design for the screening of the variables is shown in Table 1. All the variables were considered as numerical factors and investigated at two widely spaced intervals designated as −1 (low level) and +1 (high level) [19].

### 2.5. Antimicrobial Activity

The antimicrobial activity [20,21] was assessed against two spoilage microorganisms such as *A. niger* MIUG M5 and *B. subtilis* MIUG B1. Each 0.5 g of the freeze-dried fermented medium was homogenized with 45 mL of sterile Potato Dextrose Agar (PDA) or Plate Count Agar (PCA) medium, cooled to 42 °C, and poured into Petri dishes. The plates were centrally inoculated with 5 µL spore suspension with a final concentration of 1 × 10^6^ spores/mL and incubated at 25 °C for five days (for molds) and at 37 °C for 48 h (for bacteria). The control plates containing PDA and PCA media mixed with sterile distilled water in the same proportions as above were also prepared and inoculated. After the incubation period, the growth area in both treated (A_T_) and control (A_C_) plates was determined from the mean perpendicular diameter measurements by assuming a circular growth. The inhibition ratio (IR) was calculated using the following Equation (1):

(1) $$\%IR=\frac{\left(A_{C}-A_{T}\right)}{A_{C}}x100$$

### 2.6. Antioxidant Capacity

The 2,2-diphenyl-1-picrylhydrazyl (DPPH) radical scavenging activity for the freeze-dried fermented medium was performed [22]. The absorbance was read at 515 nm in a UV–Vis spectrophotometer (model Libra S22, Biochrom, Cambridge, UK) [6]. The antioxidant activity was expressed as mM Trolox equivalents (TE)/g using the Trolox (6-hydroxy-2,5,7,8-tetramethylchroman-2-carboxylic acid) calibration curve as the standard.

Previously, 1 g of the freeze-dried fermented medium was mixed with 9 mL distilled water and the extraction was performed during 30 min on an ultrasonic cleaner (MRC Ltd. Laboratory Equipment, Holon, Israel). Afterward, the samples were centrifuged at 8000 rpm for 15 min at 4 °C. The supernatant was used for further analysis.

### 2.7. Statistical Analysis

All the experiments were performed in triplicate. The results were expressed as average values with standard deviation. The statistical analysis of the data was performed using Minitab 17 (Minitab LLC, State College, PA, USA). To assess the differences between the samples and the control, the Kruskal–Wallis nonparametric test was used. The differences between samples were determined by applying the Tukey test with the one-way analysis of variance (ANOVA) method. The analysis of variance (ANOVA) for the Plackett–Burman design of the experiments was conducted using the Design Expert software version 8.0.2.0 (Stat-Ease Inc., Minneapolis, MN, USA).

## 3. Results

### 3.1. Screening of the Yeast Strains that Are Able to Produce Hydrolases

Nineteen yeast strains belonging to the MIUG Collection were tested based on their potential to hydrolase pork lard 5% (*w*/*v*) or 2% (*w*/*v*) freeze-dried okara as substrates. Two strains, coded *Y. lipolytica* MIUG D5 and *Y. lipolytica* ATCC 18942 were selected as potential producers of lipases and proteases (Table 1).

The most active strain with regard to the lipase activity was, as expected, the commercial strain coded *Y. lipolytica* ATCC 18942 with a hydrolysis ratio of 1.60 (Table 1), whereas a hydrolysis index of 1.65 was registered for the protease activity. The strain coded *Y. lipolytica* MIUG D5 showed a hydrolysis ratio of 1.20 and 1.30 for both enzymatic activities of lipase and protease activity, respectively (Table 1).

**Figure 1:**
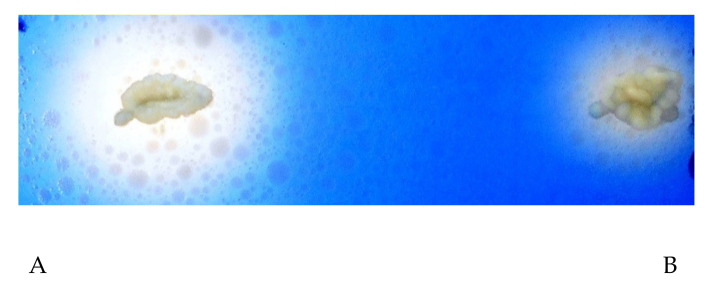
Lipase activity on spirit blue agar medium of *Y. lipolytica* MIUG D5 (**A**) and *Y. lipolytica* ATCC 18942 (**B**) strains.

Furthermore, the most active strains were tested on the spirit blue agar medium supplemented with 2% pork lard at 28 °C for 48 h. As can be seen in Figure 1, the strains coded *Y. lipolytica* MIUG D5 marked a higher lipolysis index (2.64) compared to the commercial strain coded *Y. lipolytica* ATCC 18942, which revealed a lower hydrolysis index (1.73).

### 3.2. Antimicrobial Activity

Furthermore, different fermented products were obtained considering the valorization of the freeze-dried okara and pork lard through SSF fermentation. The process took place using a single culture of *Y. lipolytica* and a co-culture of *Y. lipolytica* and *L. paracasei* in order to obtain a fermented product with antioxidant and antimicrobial activity. The inhibitory activity of the fermented products was established against spoilage mold and spore forming bacteria strains by calculating the inhibition ratio (Table 2).

The strongest antimicrobial spectrum was obtained against *B. subtilis* MIUG B1. From the data shown in Table 2, it can be observed that the fermented product obtained with the co-culture of *Y. lipolytica* ATCC 18942 and *L. paracasei* MIUG BL20 showed the highest inhibition potential against *B. subtilis* MIUG B1 (33%). Generally, the fermented products obtained with the co-culture indicated a higher antibacterial potential compared to the fermented products that were obtained with the *Y. lipolytica* monoculture. Instead, for *A. niger* MIUG M64, a lower antimicrobial activity (6–9%) and spore inhibition were observed for all the tested variants.

### 3.3. Antioxidant Activity

The antioxidant activity of the fermented products was evaluated by the DPPH radical scavenging activity method. Practically, the fermentation with the *Y. lipolytica* monoculture increased the functional quality of the fermented products compared to the products obtained with the co-culture of yeast and LAB (Table 3).

The fermented product developed with the single culture of *Y. lipolytica* MIUG D5 (1 × 10^7^ CFU/g) expressed a much higher antioxidant potential, of 18.49 ± 0.37 mM TE/g, the value being 2.69 times higher than the value of the control sample (unfermented media).

Since the data were not normally distributed (samples and control), and the *p*-value was less than 0.005, the Kruskal–Wallis nonparametric test was performed. A *p*-value of 0.011 was obtained, which means that there were significant differences between the data (samples and control). Overall, the differences between the samples were normally distributed (*p* > 0.05) since the variants were equal (*p* > 0.05) and an ANOVA *p* value of 0.002 was calculated. The data group was appreciated by applying the Tukey test with the one-way ANOVA method. The samples that do not share a letter were significantly different (*p* < 0.05) according to the ANOVA test (Table 3).

### 3.4. Screening of the Fermentation Parameters Using Plackett–Burman Design

Taking into account the results and the interesting approach of using a co-culture, a fermented product obtained with the co-culture formed of *Y. lipolytica* MIUG D5 and *L. paracasei* MIUG BL20 was developed. In order to select the significant factors that influence the functional properties of the obtained fermented product, a Plackett–Burman experimental design was performed. The values of the independent parameters were set within the Plackett–Burman design, taking into account the growth conditions of the studied microorganisms. The experiment was conducted in 12 runs and seven parameters were analyzed, whereas the rest of the parameters were aliased. Table 4 shows the results of the screening experiments using the Plackett–Burman Design.

According to the design matrix, the substrates for the culture media were varied as follows: 10–20% freeze-dried okara, 1–3% pork lard, and 0.5–1.5% glycerol. Afterward, the media were inoculated with two different concentrations of yeast strain (1 × 10^5^–1 × 10^7^ CFU/g) and incubated between 48–96 h, at 30 °C. After that, 1% and 2% of *L. paracasei* MIUG BL20 inoculum were added and incubated further for a 24–72 h period at 30 °C. At the end of the fermentation, the fermented medium was subjected to freeze-drying and then the antioxidant and antimicrobial activity were assessed. The results presented in Table 4 indicated that the obtained fermented products based on the co-culture of *Y. lipolytica* MIUG D5 and *L. paracasei* MIUG BL20 displayed an antioxidant potential ranging from 6.77 up to 17.78 mM TE/g and an antimicrobial activity against *A. niger* MIUG M5 between 24–64% inhibition ratio. The antimicrobial activity against *B. subtilis* MIUG B1 was not analyzed since a total inhibition ration (100%) was achieved. The statistical analysis of the responses is represented in Table 5 and Table 6.

The model’s *F*-value of 7.85 implies that the model was significant. The *p* ˂ 0.05 values indicated that the model terms were significant. In this case, the time of the yeast fermentation (E) is a significant model term (Table 5). The values greater than 0.05 indicated that the model terms were not significant.

The ANOVA analysis revealed a model *F*-value of 5.71, which suggested that the model was significant. In the case of the antioxidant activity of the fermented product, the concentration of pork lard (B) was considered as a significant model term with *p* ˂ 0.05 (*p* = 0.251) (Table 6).

## 4. Discussion

The aim of this study was to valorize some agri-food processing by-products such as okara [1] and pork lard [9] by applying an inexpensive and conventional biotechnological method [1], namely solid state fermentation (SSF) using a co-culture of *Y. lipolytica* and *L. paracasei* in order to obtain several functional fermented products that have been proposed for use in the food and feed industry [2,6]. These fermentative substrates were chosen because of the annually tons of okara that are generated, especially in Asian countries, and tons of pork lard that are generated in industrialized countries from Europe.

The solid-state fermentation using the GRAS recognized *Y. lipolytica* and *L. paracasei* [6,13], the nutritional and functional values of the okara and pork lard [4,10], may be significantly improved [1] by generating bioactive peptides and organic acids including fatty acids with antimicrobial potential [3]. To the best of our knowledge, no information has been found in the literature about the conversion of both okara and pork lard through SSF fermentation using a co-culture of *Y. lipolytica* and *L. paracasei*. Therefore, in our study, first, the okara and pork lard were subjected to proteolysis and lipolysis by the extracellular enzymes synthesized by the selected yeast strains, in order to obtain small molecules [23], and afterward, the *L. paracasei* strain could easily metabolize this type of molecules. In order to identify the potential yeast strains that are able to produce lipase and protease, the selection of the strains on specific substrates (5% pork lard and 2% freeze-dried okara) was accomplished.

Our results are in good agreement with the data that reported that the non-conventional *Y. lipolytica* yeast was able to produce extracellular lipases and proteases [17,24]. The first lipase obtained by converting pork lard for biorefinery was reported by Lopes et al. (2018) [9]. Additionally, Szotkowski et al. (2019) [7] studied four red yeast (*Cystofilobasidium macerans* CCY 10-1-2, *Rhodotorula glutinis* CCY 20-2-26, *R. mucilaginosa* CCY 19-4-6, and *Sporobolomyces pararoseus* CCY 19-9-6) that were able to produce an extracellular lipase by using animal fat as a substrate. Moreover, another study also described other red yeast strains (*Rhodotorula* spp., *Sporobolomyces* spp., *Cystofilobasidium* spp.) that are able to produce polyunsaturated fatty acids from animal fat waste [25].

From our previous results, several yeast strains were identified as active producers of lipase and protease by using bovine colostrum and cow milk fat as substrates [26,27,28]. *Y. lipolytica* A-101 and W29 produced a lipase after 54 h of incubation at 25 °C (2.3 mm) on the spirit blue agar medium supplemented with butter fat [29].

The antimicrobial activity of the fermented products could be correlated with the organic acids produced by the LAB and yeast strains that are responsible for the growth inhibition of the aerobe spore forming microorganisms. Hence, the antimicrobial potential of the fermented products could be associated with the production of organic acids, also including small chain saturated fatty acids, peptides (bacteriocins), carbon dioxide, hydrogen peroxide, ethanol, and diacetyl [30]. Our previous results stated that the fermented product obtained by bovine colostrum fermentation with kefir grains enhanced with a selected *Candida lypolitica* inoculum showed an inhibition zone ranging between 3.0 and 5.0 mm against *B. subtilis* MIUG B1, while for *A. niger* MIUG M64, spore inhibition was observed [26,28].

The DPPH radical scavenging activity of the fermented product derived from the principal substrates, okara and pork lard, increased during fermentation. Our results were in accordance with Patrignani et al. (2011) [31], who described that the fermented okara showed the highest DPPH radical scavenging activity of 43.68 µg TE/g dried okara after 72 h of fermentation with *B. subtilis* WX-17. Compared to the DPPH radical scavenging activity of the unfermented okara of 6.79 µg TE/g dried okara, the fermented okara displayed an increase of the DPPH radical scavenging activity by approximately 6.4 times.

Taking into account the obtained results and the novelty of this approach, the selection of the most important parameters that influence the SSF fermentation of agri-food by-product substrates using a co-culture of *Y. lipolytica* MIUG D5 and *L. paracasei* MIUG BL20 was performed. The statistical analysis highlighted that the concentration of the pork lard influenced the antioxidant activity of the fermented product, while the antimicrobial activity was determined by the time of fermentation with the selected yeasts.

The effects of temperature, water activity, and yeast inoculation level upon the lipolytic pattern and volatile production by *Y. lipolytica* Y16A, according to a central composite design (CCD) using pork fat as a substrate, was assessed by Patrignani et al. (2011) [31]. Another study underlined the effect of pH, pork lard concentration, arabic gum concentration, and oxygen mass transfer rate on the metabolic activity of the *Y. lipolytica* strain [9].

Based on the Plackett–Burman design and analysis, the most important biotechnological parameters that influence the metabolic activities of the yeasts and lactobacili cultivated in a co-culture in order to obtain a fermented product with improved antioxidant and antimicrobial activities are the time of the yeast fermentation and the concentration of pork lard.

Further experiments will take into account the optimization of the most important factors that influence the obtainment of the functional fermented product by using the CCD and the response surface methodology (RSM) tools. Then, the identification and characterization of the compounds responsible for the antimicrobial and antioxidant activity will be taken into consideration as well as organic acids including the short chain saturated fatty acids as well as some valuable bioactive peptides. The fermented product is recommended for use as a food or feed ingredient with biotic properties in order to improve the functional composition, the shelf life, and the safety assurance. From an economic point of view, the proposed biotechnological process is cheap and quite feasible for the valorization of agri-food by-products. Thus, in accordance with the principles of a circular economy, the valorization of waste into new functional bioproducts is a modern emergent task that offers new approachable applications.

## 5. Conclusions

The results of the present study demonstrated the improved functional characteristics of the okara and pork lard by SSF fermentation with a co-culture of *Y. lipolytica* and *L. paracasei*, which is an innovative approach to deal with the disposal of okara and pork lard in order to obtain value-added fermented products with the potential for use as a bioingredient in food or feed formulation. To summarize, the research targeted the screening of the most active yeast strain capable of producing lipase and protease by cultivation on these substrates. Furthermore, the fermented product obtained through the SSF fermentation of the okara and pork lard with a co-culture of *Y. lipolytica* and *L. paracasei* highlighted its valuable properties (antioxidant and antimicrobial activity). Additionally, the results allowed for the selection of the optimal solid-state fermentation parameters that offer the perspective for the optimization of the process and also for the improvement of the functional properties of the fermented product. The residual okara and pork lard that were used as a substrate for the yeasts and lactic acid bacteria provide biologically active compounds with promising applications for the development of a novel bioingredient.

## Figures and Tables

**Table 1:** Selection of the yeast strains able to produce lipases and proteases.

No.	Strain Code	Protease Assay *	Lipase Assay *
1	*Y. lipolytica* MIUG D5	1.30	1.20
2	*Y. lipolytica* MIUG D6	−**	−
3	*Y. lipolytica* MIUG D7	−	−
4	*C. lipolytica* MIUG 106	1.16	1.08
5	*C. lipolytica* MIUG D67	−	−
6	*C. lipolytica* MIUG D69	−	−
7	*C. lipolytica* MIUG D96	1.05	1.03
8	*C. lipolytica* MIUG D98	1.13	1.05
9	*C. lipolytica* MIUG D99	−	1.03
10	*C. lipolytica* MIUG D100	1.15	1.08
11	*C. lipolytica* MIUG D101	1.10	1.05
12	*C. lipolytica* MIUG D111	1.05	1.03
13	*C. lipolytica* MIUG D73	−	nd
14	*C. colliculosa* MIUG D108	−	nd
15	*C. krusei* MIUG D97	−	nd
16	*C. colliculosa* MIUG D102	−	nd
17	*C. colliculosa* MIUG D115	−	nd
18	*C. krusei* MIUG D74	−	nd
19	*Y. lipolytica* ATCC 18942	1.65	1.60

* Hydrolysis ratio after five days of cultivation at 28 °C; ** Developed colony, without clear zone; nd—undeveloped colony.

**Table 2:** Antimicrobial activity of the fermented product against *A. niger* and *B. subtilis.*

Sample	Inhibition Ratio %	
	*A. niger* MIUG M5	*B. subtilis* MIUG B1
Fermented product with *Y. lipolytica* MIUG D5	6	16
Fermented product with *Y. lipolytica* ATCC 18942	9	23
Fermented product with co-culture of *Y. lipolytica* MIUG D5 and *L. paracasei* MIUG BL20	0 *	29
Fermented product with co-culture of *Y. lipolytica* ATCC 18942 and *L. paracasei* MIUG BL20	0	33

* Sporulation was inhibited; the values are the mean of three replicates.

**Table 3:** Antioxidant activity of the fermented products.

Sample	Antioxidant Activity, mM TE/g
Control	6.86 ± 1.07 *
Fermented product with *Y. lipolytica* MIUG D5	18.49 ± 0.37 ^a^
Fermented product with *Y. lipolytica* ATCC 18942	18.06 ± 0.55 ^ab^
Fermented product with co-culture of *Y. lipolytica* MIUG D5 and *L. paracasei* MIUG BL20	17.27 ± 0.75 ^bc^
Fermented product with co-culture of *Y. lipolytica* ATCC 18942 and *L. paracasei* MIUG BL20	14.60 ± 0.30 ^c^

* The values are a mean of three replicates ± standard deviation; a–c statistical groups, samples that do not share a letter were significantly different (*p* < 0.05)

**Table 4:** Placket–Burman experimental design for the screening of the most significant variables upon Solid State Fermentation (SSF) fermentation.

Run	Independent Variables							Responses	
	A *	B	C	D	E	F	G	Antioxidant Activity, mM TE/g	Antimicrobial Activity%
1	20.00	3.00	1.50	1 × 10^5^	48.00	1.00	72.00	13.09	44
2	20.00	1.00	0.50	1 x 10^5^	96.00	1.00	72.00	6.77	28
3	10.00	3.00	1.50	1 × 10^5^	96.00	2.00	72.00	15.17	44
4	10.00	1.00	0.50	1 × 10^7^	48.00	2.00	72.00	10.75	36
5	10.00	3.00	0.50	1 x 10^7^	96.00	1.00	72.00	17.78	44
6	10.00	1.00	1.50	1 × 10^5^	96.00	2.00	24.00	14.06	24
7	20.00	1.00	1.50	1 × 10^7^	96.00	1.00	24.00	13.45	32
8	10.00	1.00	0.50	1 × 10^5^	48.00	1.00	24.00	11.28	40
9	20.00	3.00	0.50	1 × 10^5^	48.00	2.00	24.00	8.95	40
10	20.00	3.00	0.50	1 × 10^7^	96.00	2.00	24.00	13.36	44
11	20.00	1.00	1.50	1 × 10^7^	48.00	2.00	72.00	13.31	64
12	10.00	3.00	1.50	1 × 10^7^	48.00	1.00	24.00	12.11	34

* A—freeze-dried okara (*w*/*v*%), B—pork lard (*w*/*v*%), C—glycerol (*w*/*v*%), D—yeast inoculum (CFU/g), E—time for yeast fermentation (h), F—LAB inoculum (%), G—time co-fermentation (h).

**Table 5:** Analysis of variance (ANOVA)of the effect of the studied parameters upon the antifungal activity of the fermented products.

Source	Sum of Squares	df	Mean Square	*F* Value	*p*-Value
Model	507.00	1	507.00	7.85	0.0187
E-time for yeast fermentation	507.00	1	507.00	7.85	0.0187
Residual	646.00	10	64.60	/	/
Cor Total	1153.00	11	/	/	/

**Table 6:** ANOVA analysis of the effect of the studied parameters upon the antioxidant activity of the fermented products.

Source	Sum of Squares	df	Mean Square	*F* Value	*p*-Value
Model	50.44	2	25.22	5.71	0.0251
B-pork lard	38.52	1	38.52	8.71	0.0162
F-LAB inoculum	11.92	1	11.92	2.70	0.1350
Residual	39.78	9	4.42	/	/
Cor Total	90.23	11	/	/	/
